# Medical Food Assessment Using a Smartphone App With Continuous Glucose Monitoring Sensors: Proof-of-Concept Study

**DOI:** 10.2196/20175

**Published:** 2021-03-04

**Authors:** Hector Roux de Bézieux, James Bullard, Orville Kolterman, Michael Souza, Fanny Perraudeau

**Affiliations:** 1 Pendulum Therapeutics, Inc San Francisco, CA United States; 2 Division of Biostatistics School of Public Health University of California, Berkeley Berkeley, CA United States; 3 Center for Computational Biology University of California Berkeley, CA United States

**Keywords:** clinical trials, continuous glucose monitoring, lifestyle modification, mobile app, telemedicine, diabetes

## Abstract

**Background:**

Novel wearable biosensors, ubiquitous smartphone ownership, and telemedicine are converging to enable new paradigms of clinical research. A new generation of continuous glucose monitoring (CGM) devices provides access to clinical-grade measurement of interstitial glucose levels. Adoption of these sensors has become widespread for the management of type 1 diabetes and is accelerating in type 2 diabetes. In parallel, individuals are adopting health-related smartphone-based apps to monitor and manage care.

**Objective:**

We conducted a proof-of-concept study to investigate the potential of collecting robust, annotated, real-time clinical study measures of glucose levels without clinic visits.

**Methods:**

Self-administered meal-tolerance tests were conducted to assess the impact of a proprietary synbiotic medical food on glucose control in a 6-week, double-blind, placebo-controlled, 2×2 cross-over pilot study (n=6). The primary endpoint was incremental glucose measured using Abbott Freestyle Libre CGM devices associated with a smartphone app that provided a visual diet log.

**Results:**

All subjects completed the study and mastered CGM device usage. Over 40 days, 3000 data points on average per subject were collected across three sensors. No adverse events were recorded, and subjects reported general satisfaction with sensor management, the study product, and the smartphone app, with an average self-reported satisfaction score of 8.25/10. Despite a lack of sufficient power to achieve statistical significance, we demonstrated that we can detect meaningful changes in the postprandial glucose response in real-world settings, pointing to the merits of larger studies in the future.

**Conclusions:**

We have shown that CGM devices can provide a comprehensive picture of glucose control without clinic visits. CGM device usage in conjunction with our custom smartphone app can lower the participation burden for subjects while reducing study costs, and allows for robust integration of multiple valuable data types with glucose levels remotely.

**Trial Registration:**

ClinicalTrials.gov NCT04424888; http://clinicaltrials.gov/ct2/show/NCT04424888.

## Introduction

Diabetes is one of the most prevalent chronic diseases in the world, impacting over 422 million people worldwide [1]. In the United States alone, type 2 diabetes (T2D) affected over 32 million individuals as of 2018 [2]. Western lifestyle, including diet, has been shown to play a clear role in the development of the disease [3-5]. Although a large and growing number of drugs have been approved for the treatment of T2D, no cure or universally effective pharmacological intervention yet exists.

By contrast, studies where sustained dietary and behavioral changes were made (eg, caloric restriction) have shown profound improvements in glucose control in subjects who are able to adhere to the regimen [6]. For example, in a study conducted by Lean et al, 306 individuals with T2D were given an 850-calorie diet. While only 24% achieved the primary end goal of weight loss of ≥15 kg, 86% of subjects who achieved the goal saw remission of their diabetes by 12 months [7]. Such low compliance, which is routinely observed in trials with dietary and behavioral changes, limits the interpretability of the intervention. In addition, rigorously accounting for such attrition in a randomized controlled trial (RCT) can dramatically increase the cost and complexity of conducting such studies, especially over long periods of time. As a consequence, rigorous trials of feasible dietary changes in routine clinical practice settings are frequently not performed, which in turn limits the body of clinical evidence available to guide treatment [8,9]. Studies that are less invasive in terms of the participants’ usual daily activities may enable collection of endpoint measurements matching the quality of traditional clinical study assessments while decreasing dropouts and improving the tracking of compliance.

The growing use of mobile technologies, wearable biosensors, and telemedicine in RCTs allows for closer patient monitoring and increased engagement while limiting overhead [10]. If a sensor can provide high-quality measurements of relevant clinical metrics, it may be leveraged to conduct robust trials outside the traditional clinical trial setting. In the context of diabetes, continuous glucose monitoring (CGM) devices represent a widely used type of sensor. A new generation of devices [11] has overcome earlier drawbacks of previous CGM devices, including cost and the continuing need for calibration [12], and has gained regulatory approval [13-15]. Consequently, the number of clinical trials using CGM devices to measure primary or secondary endpoints has increased [16-22], permitting new innovative approaches and clinical trial designs. For example, in a previous report [23], 36 subjects were enrolled remotely and received CGM devices via shipments. Instructions on CGM application and usage were given remotely, and 34 subjects used the devices correctly more than 95% of the time. Such recent results may enable and support the conduction of robust investigations outside the usual clinical trial setting.

Although CGM deployment in clinical research is now well established, the advent of this new generation of glucose monitors has led to a large increase in studies relying mostly or solely on CGM data as primary outcome measures. Despite this, most studies do not fully leverage the potential of CGM devices, as they are deployed only for specific time periods in the study, for example, the first and last days of the trial [17-21], and when conducting interventions and monitoring while attending a clinical center [24,25]. Furthermore, integrating and augmenting the data provided by CGM devices with other data types, such as food logs and activity data, are still relatively unexplored.

In this manuscript, we present the results from a 6-week, double-blind, randomized, placebo-controlled, exploratory 2×2 cross-over study (n=6). A custom smartphone app was used to record food intake, exercise, and alcohol consumption and integrate these data with CGM data to construct a visual diet log in sync with concurrent glucose levels. In a concomitant study [26], we observed changes in postprandial glucose response in patients with T2D who consumed a five-strain synbiotic medical food manufactured by the study sponsor. The postprandial glucose response was measured following a standardized meal-tolerance test (MTT) administered at the clinic. In parallel, we were also interested in following subjects using the medical food while they pursue their daily activities unencumbered by the disruptions introduced by required visits to a research clinic. Thus, in this exploratory study, subjects consumed the medical food, and the postprandial glucose response was measured using CGM devices via a self-administered MTT at home. The aim of this study was to demonstrate the feasibility of measuring clinically relevant data for research purposes using CGM devices and a smartphone app while subjects pursue their normal daily activities.

## Methods

### Study Design

A double-blind, randomized, placebo-controlled cross-over design was used to compare responses to the following two experimental interventions: a synbiotic medical food product, which was provided by the study sponsor, in the form of three capsules taken twice a day, and an inactive placebo present in similar capsules (Multimedia Appendix 1). Subjects wore CGM (Freestyle Libre) devices through the entirety of the study according to the manufacturer’s instructions for use. At baseline, beginning/end of each period, and washout, subjects were asked to collect a stool sample. The concentrations of the bacterial strains, which were ingested in the medical food, in those stool samples were estimated using quantitative polymerase chain reaction (qPCR). Strain concentrations were averaged across three replicates. One subject (Subject 4) did not provide stool samples, so this part of the analysis was performed in five subjects. Anthropometric characteristics were also measured.

Throughout the study, subjects used a custom smartphone app for the Android platform. This app was developed to facilitate the real-time collection and integration of glucose levels, a visual food log, an alcohol log, and an exercise log, as well as record scanning compliance (how often do subjects scan their CGM devices) and study events (such as MTT). Through the app, subjects could take pictures and annotate them to describe their food consumption. They could also submit text annotations without pictures to log events such as exercise. Screenshots of the mobile app can be found in Multimedia Appendix 2 and Multimedia Appendix 3.

The goal of this exploratory study was to determine how to collect and analyze appropriate glucose metrics when conducting a medical food study with CGM devices and a novel smartphone app. This includes measuring operating characteristics (sensitivity and specificity) and sources of variance for those metrics when determined with Freestyle Libre glucose sensors, as well as identifying proper statistical tests of glucose metrics, for example, to test for changes in areas under the curve (AUCs) for glucose derived from self-administered MTTs between active and placebo groups. Additional endpoints of interest were change in body mass, change in fecal probiotic strain concentration, expected lifespan of CGM sensors, CGM sensor scanning, photo logging of compliance rates, and usability feedback.

Subjects were randomly assigned to the two study arms (study product then placebo or vice versa). Subjects were assigned in pairs so that each arm contained the same number of subjects. Study staff and subjects were blinded to the identity of the study product. Randomization was performed by a statistician not directly involved in study conduct. Both periods lasted 13 to 17 days and were separated by a washout period of 3 to 6 days. Details of the durations of the study periods for each subject can be found in Multimedia Appendix 4.

This study was approved on March 13, 2018, by the Aspire Institutional Review Board (IRB) (protocol number WB01-205). Informed consent was obtained from each subject prior to participation. Since it was a prospective study, it was retrospectively registered on ClinicalTrials.gov (registration number NCT04424888).

### Subject Enrollment and Characteristics

Since this was an exploratory study, the main inclusion criterion was a minimum age of 17 years. Key exclusion criteria were pregnancy; continuous parenteral nutrition; and current use or planned use of antibiotic, antifungal, antiparasitic, or antiviral treatment during the study. Subjects were recruited by word of mouth from members of the biotechnology research community in San Francisco, California. A total of six subjects were enrolled in the study. Subjects were remunerated in the form of an Amazon gift card of US $100 for successful completion of each of the two study periods. Subjects had a run-in of 3 to 5 days before the first period (product/placebo) to familiarize themselves with the CGM devices and demonstrate that they could employ the devices as instructed. Subjects were shown how to use their CGM devices and how to log their meals and exercise and take pictures of their food intake through the smartphone app. Throughout the baseline period, they could ask questions about CGM usage. All subjects mastered correct use of the CGM devices and the smartphone app under observation by the study coordinator at the clinic. This consisted of properly collecting 24 hours of data and demonstrating the ability to take pictures and make annotations through the app.

### Data Collection

**Figure 1:**
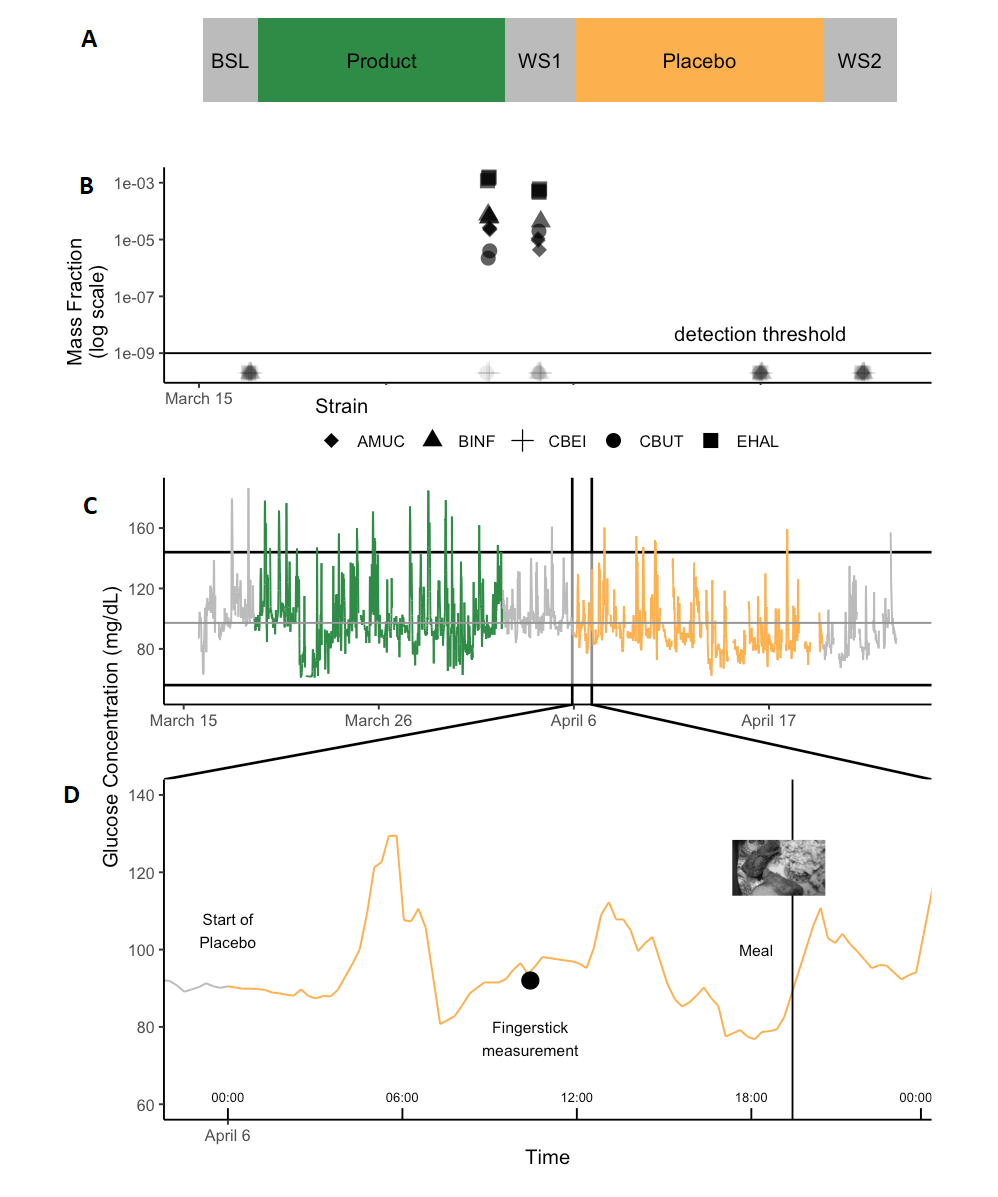
Glucose and dietary data collected for one subject. All data types collected for one subject. (A) Subject 1 starts in the active product arm (14 days) and then moves to the placebo arm (14 days). (B) At baseline, the end of each period, and study end, the subject provides stool samples. Strains present in the product are detected at the end of the active product period and a little after (arrow mark). (C) Continuous glucose monitoring (CGM) glucose levels are also tracked throughout the 6 weeks. (D) The values can be compared to the fingerstick measurements or used in coordination with pictures taken (here chicken wings at 7 PM) to detect meal-related glucose excursions. AMUC: *Akkermansia muciniphila*; BINF: *Bifidobacterium infantis*; BSL: baseline; CBEI: *Clostridium beijerinckii*; CBUT: *Clostridium butyricum*; EHAL: *Anaerobutyricum hallii*; WS: washout.

The Freestyle Libre CGM devices used in this study provide estimates of interstitial fluid glucose levels. At 15-minute intervals, the estimates are recorded in the device and stored for up to 8 hours. Whenever the CGM receiver is wanded over the device, the 8 hours of data in the device at that time are downloaded for storage in an external database. These data can be used to display a subject’s glucose levels at various time intervals. For a subject who started with the study product followed by washout (WS1), crossed over to placebo, and ended with a second washout (WS2) (Figure 1A), for example, we can visualize the glucose level throughout the study (Figure 1C) or zoom in on a specific day (Figure 1D). Glucose levels as estimated by the CGM device can be complemented by other data sources. A visual food log was constructed as subjects logged pictures of their meals through the smartphone app. As evidenced in Figure 1D, the recorded meal was associated with a spike in glucose levels. To facilitate interpretation of CGM data, we developed a peak detection algorithm based on methodology from Palshikar [27] that can automatically detect glucose peaks (Multimedia Appendix 1).

To ensure that their CGM devices were appropriately inserted, subjects also used conventional fingerstick devices (FreeStyle or CVS Health Blood Glucose Meter) to measure capillary glucose levels several times during the study and reported the values through the app. Fingerstick and CGM measurements were in good agreement (Multimedia Appendix 5) and were within the expected range [13].

At the end of the study, after unblinding, the clinical coordinator conducted an interview with all subjects, excluding Subject 5. This interview was guided by a predefined template (Multimedia Appendix 6). Questions focused on the following categories: study design, product, CGM device, mobile app, behavior changes, future studies, and closing remarks, and there was a mix of open-ended and closed-ended questions. Subjects were also asked if they would participate in another similar study and if they would recommend others to a study like this, on a scale ranging from 1 (no) to 10 (absolutely).

Finally, to assess the presence of our strains, stool samples were collected throughout the study, and strain concentrations were measured by qPCR of frozen fecal samples. qPCR analysis was conducted in five of the six subjects who provided stool samples. In Figure 1B, strain concentration information is overlaid with arm assignment. As expected, we only detected the presence of the strains after study product administration.

### Measuring the Response to a Fasted MTT

The fasted MTT is a gold standard test to measure glucose control in subjects. A standardized meal was to be consumed in the morning after fasting for at least 6 hours, and subjects were asked to ingest no additional calories for one additional hour. All subjects ingested the same standardized meal as follows: a Boost nutritional drink containing 45 g of carbohydrates including 22 g of sugars [28]. The MTT was performed four times throughout the study by each subject (at the start and end of both the placebo and product periods).

Subjects recorded when they ingested the Boost drink through the smartphone app, and corresponding glucose levels were obtained from the CGM devices. The incremental AUC for glucose levels was calculated for each time point and subject [29]. The baseline for the incremental AUC was defined as the glucose level at *t*=0, when the Boost ingestion began, as recorded by the CGM device.

The AUCs at the beginning and end of the placebo/product periods were compared using ΔAUC as follows:

ΔAUC = AUC_End_ – AUC_Beginning_**(1)**

Finally, the cross-over design allowed the comparison of ΔAUC between the placebo and active products (ΔΔAUC). We performed a one-sided Wilcoxon signed-rank test to test the null hypothesis (H0: ΔΔAUC ≥0 versus H1: ΔΔAUC <0).

## Results

### Subjects and Sensors

All six enrolled subjects completed the study. No adverse events or major deviations from the protocol were reported. All hypoglycemic events (defined as excursion below 70 mg/dL for at least two consecutive data points, ie, 30 minutes) are summarized in Multimedia Appendix 7, according to the recommendations of Schnell et al [30]. In accordance with manufacturer recommendations, CGM sensors were worn for an average of 13 consecutive days. The first sensor was placed under the supervision of the study coordinator. Subjects then placed their second and third sensors by themselves. Four subjects (1, 3, 5, and 6) had to replace one sensor each after only 10 days, mostly because the sensor was dislodged while dressing. Multimedia Appendix 8 recapitulates a few glucose-based subject-level metrics, according to the report by Schnell et al [30].

During interviews, subjects reported that CGM usage was a positive experience (Multimedia Appendix 9). However, two subjects inadvertently dislodged sensors because of clothing snags. Two subjects also reported minor soreness shortly after CGM implantation.

### Fasted MTT Using CGM Devices

At four time points throughout the study, subjects performed MTTs as described above. MTTs were performed just before the start and at the end of the placebo/product periods. The standardized MTT data were used to compare a potential change in the glucose response for a fixed nutrient intake. If the study product had the effect of improving a subject’s glucose metabolism, a decrease in the peak glucose level and/or AUC would be expected.

**Figure 2:**
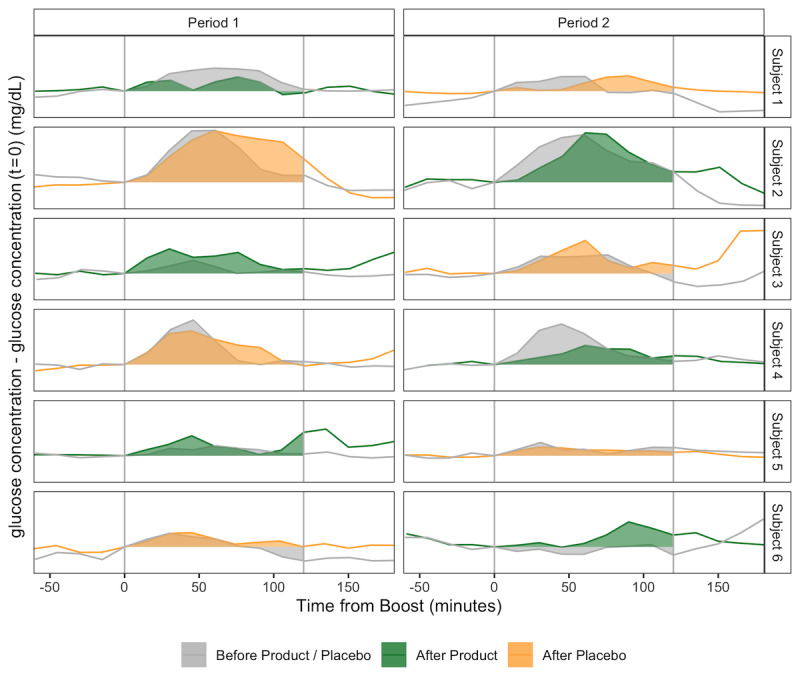
Results of the meal tolerance test (MTT). Glucose response to standardized meals for all six subjects in the study. Area under the curve values for the beginning of placebo/product, end of placebo, and end of product periods are in gray, yellow, and green, respectively. The MTTs are annotated by the subjects at time *t*=0 using the smartphone app. Glucose levels are normalized by the glucose level at *t*=0.

As shown in Figure 2, most subjects displayed an increase in glucose levels after taking the standardized meal. Each individual performed four MTTs (one at the beginning of each period, one at the end of the placebo period, and one at the end of the product period). Since the CGM devices used in this study generated a glucose measurement every 15 minutes, the curves are quite smooth, which allowed a robust computation of the AUC. The MTT data were obtained without additional subject engagement other than ingesting the standardized meal at home.

For Subjects 1, 2, and 4, the AUC decreased on ingesting the active study product, and ΔAUC_Product_ was smaller than ΔAUC_Placebo_. For Subjects 3, 5, and 6, the AUC increased on ingesting the product, and ΔAUC_Product_ was greater than ΔAUC_Placebo_ (Table 1 and Multimedia Appendix 10). Owing to the scale (n=6) and design of this pilot study, there is very limited power. In addition, none of the subjects were diagnosed with T2D, so we do not expect to find statistically significant results. Accordingly, the one-sided Wilcoxon signed-rank test had a *P* value of .28.

**Table 1:** Subjects ranked by baseline value of area under the glucose curve.

Subject^a^	Baseline AUC^b^ (mg/dL/min)	ΔAUC_Placebo_ (mg/dL/min)	ΔAUC_Product_ (mg/dL/min)	ΔΔAUC (mg/dL/min)
6	265.3	1035.1	1683.2	648.1
3	737.0	1027.1	1598.3	571.2
5	834.8	102.8	552.8	450.0
4	1557.6	427.7	−933.0	−1360.7
1	2084.2	−221.6	−1213.9	−992.3
2	3931.8	1309.1	−978.1	−2287.2

^a^For Subjects 1, 2, and 4, the AUC decreased when taking the product and ΔAUC_Product_ was smaller than ΔAUC_Placebo_.

^b^AUC: area under the curve.

As this was a small pilot study, we did not use any baseline metric to stratify subjects based on their initial glucose control levels. However, a post-hoc analysis revealed that baseline AUC stratified the subjects into two clear and distinct groups (Table 1). Interestingly, those groups exactly matched the responder (AUC decrease)/nonresponder (AUC increase) status. Baseline AUC is an indication of the subject’s glucose control level at the onset of the study. This is an interesting observation that should be explored in follow-up studies designed and powered appropriately. Using the mean ΔΔAUC value of the three responders as a reasonable effect size and considering the variance of the ΔΔAUC value from all subjects, a future study employing the same study design with 35 subjects is estimated to have a power above 90%.

### CGM Enables Novel Measures of Compliance

**Figure 3:**
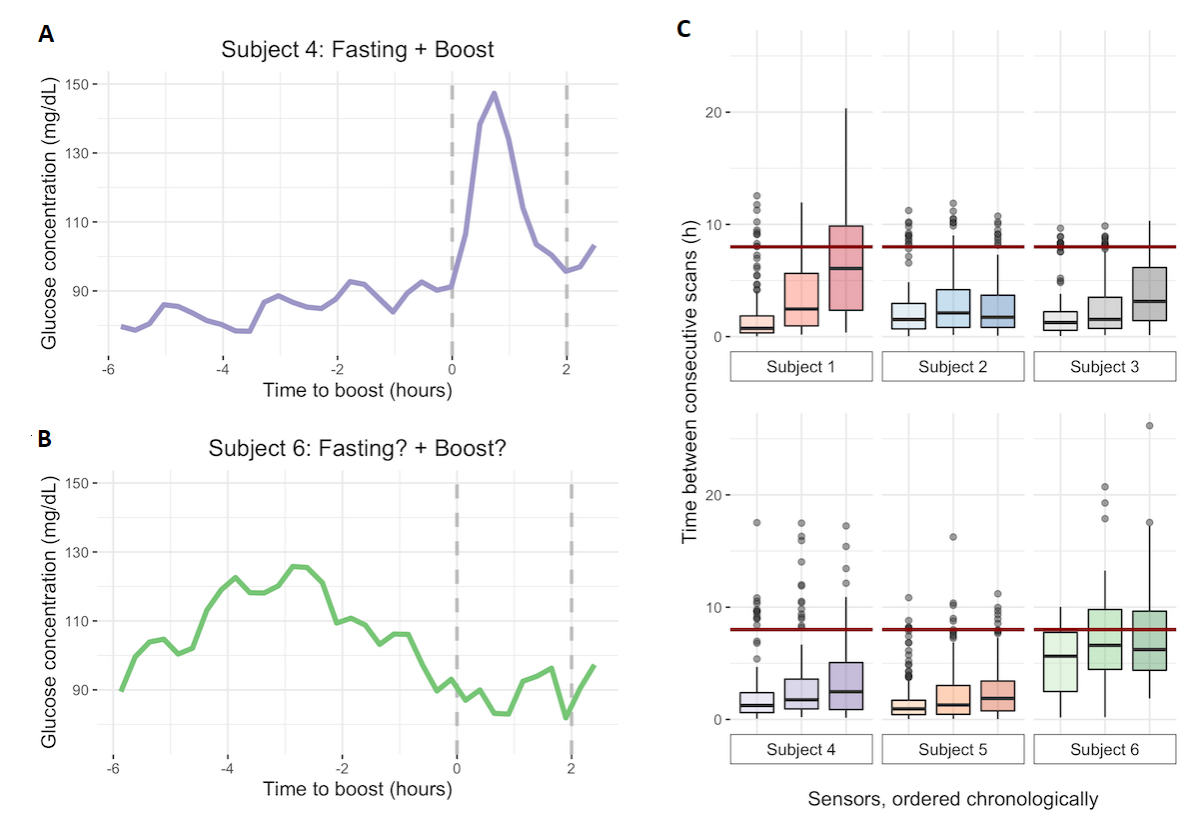
Continuous glucose monitoring (CGM) devices provide insight into compliance. (A) CGM data are concordant between when Subject 4 mentioned fasting/consuming the standardized meal for the glucose challenge and what was detected from the CGM device. (B) CGM data can also be used to detect anomalies. There is no concordance between when Subject 6 recorded fasting/consumption of the standardized meal and what was observed from the CGM data. (C) Each subject is asked to obtain data at regular intervals and at least every 8 hours (red line), which is the limit for the sensor memory to store data.

CGM devices can be utilized to perform MTTs in the subject’s living environment instead of the artificial setting of a research clinic. In this study, subjects were asked to use a custom Android app to create a visual food diary and log workouts and the start times of four standardized meal consumption tests. As such, we know precisely when the MTT was conducted. Subjects were asked to fast for 6 hours before taking the MTT, and Subject 4 seemed to have adhered to this. Indeed, the glucose level curve of this subject was rather flat for the 6 hours preceding the test. Then, right after ingesting the standardized meal, the glucose level spiked in a characteristic manner before returning to the baseline level after 2 hours (Figure 3A). On the other hand, Subject 6’s response was quite different. For the test at the beginning of the product period, Subject 6’s glucose level curve did not stay flat for the 6 hours before the glucose challenge (Figure 3B). Instead, it spiked from 90 mg/dL to 130 mg/dL and back over the course of 5 hours. Afterwards, glucose levels stayed flat during the MTT. Several explanations are possible. The MTT was performed at 7 AM, so the preceding peak might reflect a dawn effect [31], poor quality of sleep, alcohol consumption during the previous evening, or nighttime snacking. The subject’s last recorded annotation before the Boost intake was at 8.44 PM the previous evening, so there is no indication of what could have caused this. Nonetheless, the fact that such an event was detectable before the MTT was performed is valuable information. Indeed, in such a case, it is easy to probe the subject for additional details to understand potentially confounding circumstances and ask the subject to take the MTT again the following day. This would lower variability by improving the quality of the MTT data, resulting in a more precise estimate of the product effect. A single estimate of fasting glucose at *t*=0, either via a fingerstick measurement or a blood draw as traditionally performed in clinical trials, would have failed to detect this.

A CGM device could also serve as an easy and efficient device for measuring engagement in the study since subjects needed to scan their devices regularly. In particular, since the devices used in the study could only store 8 hours of data, consecutive scans were not supposed to be more than 8 hours apart. Measuring the time intervals between scans and comparing them to the 8-hour mark provides a measure of the subject’s compliance. In Figure 3C, time intervals between all consecutive scans are displayed. As the study progressed, all but one subject tended to let more time go between scans. This is consistent with interviews where subjects reported fatigue as the study progressed. Moreover, the number of scans with more than 8 hours in between also increased. This expected study fatigue affected subjects differently. While Subjects 3, 4, and 5 had small increases in their median times between scans, Subject 2 actually saw a small decrease by the end of the study. Subject 1 went from being the most compliant to the least over the course of the study, whereas Subject 6 displayed a relative lack of compliance throughout the study. Access to these data in real time could guide and target efforts to enhance compliance where needed.

Subjects could also record pictures and annotations through the smartphone app. The number of logs every day varied greatly between subjects, where the average number of logs per day ranged from 2.9 for Subject 4 to 9.73 for Subject 5, and throughout the study, as the variance for the number of logs per day was around 41% of the average. This reflects various behaviors that are captured by the smartphone app and that complement the CGM devices. For example, Subject 4 was one of the most compliant in terms of scanning requirements but logged the fewest number of meals. Study fatigue was also far less apparent than that in scanning requirements, as the average number of annotations per day decreased only slightly with time, suggesting that engagement with the smartphone was easier to maintain. However, compliance was also impacted by social norms, as reported in interviews. Subjects were less likely to log pictures in social settings and thus annotated after the fact, although recent trends, such as posting pictures of meals on social networks, have lowered the barrier.

### Integrating CGM Data With the Smartphone App

With a measurement every 15 minutes for 6 weeks, the CGM devices produced about 3000 data points per subject throughout the study. An MTT has nine CGM measurements (a measurement every 15 minutes for 2 hours), so all four MTTs represented a total of 36 points per subject throughout the study (1% of the data collected). Here, we show that the CGM devices do capture a trove of information that is not available in current clinical designs without CGM devices.

One feature of interest in continuous monitoring of glucose levels is glucose peaks. Those peaks are induced by a diverse set of behaviors, mostly food consumption and, in some subjects, exercise. Glucose peaks are characterized by their range and duration. Glucose levels of 140 mg/dL or above 2 hours after a meal are indicative of a lack of glucose tolerance [32]. Using our peak detection algorithm, we found that the subjects spent an average of 5 hours and 47 minutes of their day in glucose peaks, representing roughly 25% of all the data collected, whereas MTTs comprised only 1% of the data. We detected between two and three peaks a day, consistent with a routine of three meals a day.

**Figure 4:**
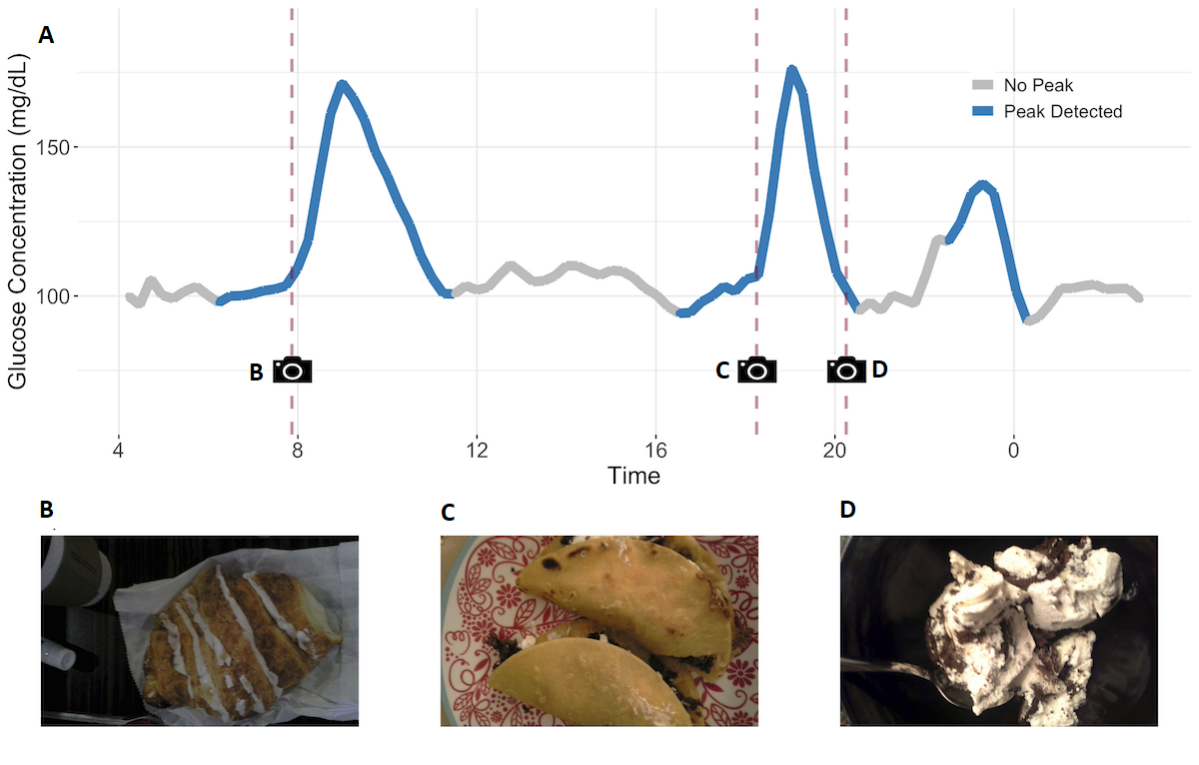
Continuous glucose monitoring (CGM) devices capture more data than the standard meal-tolerance test. (A) Zooming on a specific day allows one to display an entire day of glucose estimates (March 20, 2018, for Subject 1). Peaks are detected with the in-house algorithm (Multimedia Appendix 1) and colored in blue. Annotations logged in the smartphone app by the subject are marked with a dashed red vertical line and a picture logo. (B-D) Pictures submitted by Subject 1 through the app on March 20, 2018.

An example of the types of peaks detected by the algorithm can be seen in Figure 4, where the results for March 20, 2018, for one subject are shown. The algorithm detected three peaks during this day, clearly differentiating between noisy fluctuations of glucose levels and glucose peaks induced by a meal. While most peaks are easy to distinguish visually, the amount of data means that automated annotation is necessary.

Integrating the CGM data with the visual diet log provides rich and insightful information. In particular, since the smartphone app was used for both recording glucose levels and taking pictures, we can precisely match the times of one with the other. On March 20, 2018, the subject recorded three meal pictures (Figure 4B-D). The start of the first and second peaks matched very well with the pictures taken. Interestingly however, the third picture (Figure 4D) and the third peak did not match. This picture was recorded over an hour before the start of the next glucose peak. The high fat content visible in the meal (Figure 4D) is consistent with this delay.

The app was only available for Android-based devices, and for this exploratory study, subjects were provided with compatible smartphones. The need to carry a second smartphone was highlighted as a burden in interviews, especially by users of alternative smartphone operating systems. Development of a general app that can be used directly on a subject’s smartphone would lessen the burden on subjects in a future study. Subjects also mentioned several improvements to the design that would facilitate their interactions with the app.

### qPCR-Based Secondary Endpoints

At baseline, the beginning/end of each period, and washout, subjects were asked to provide a stool sample. Analysis of stool samples was performed in five of the six subjects; one subject did not provide stool samples. Using fecal qPCR, the concentrations of the strains in the formulation were estimated. In the results shown in Multimedia Appendix 11, strain concentrations were averaged across three replicates. As expected, at baseline, during placebo, and during placebo washout, the strains in the formulation were not detected. This was true even when the placebo period followed the product period. Most strains were detected at the end of the product period in all subjects (Multimedia Appendix 11). For the subjects with a washout duration of 1 to 3 days (Multimedia Appendix 4), the strains in the formulation could still be detected, although generally at lower levels, after the product washout, suggesting that a washout period of 3 days is too short. Additionally, for the subjects on the active product during period 1 (Subjects 1, 3, and 5), strains were not detected at the end of the placebo period, suggesting that a washout duration between 5 days and 2 weeks is sufficient.

### Code and Data Availability

All the codes and data necessary to reproduce the figures and tables in this paper are available at GitHub [33].

## Discussion

### Principal Findings

This manuscript describes a pilot study exploring the potential of CGM devices with a smartphone app that collects additional data as an avenue for measuring clinically relevant endpoints, assessing compliance in realistic settings, and decreasing the burden of study participation while collecting valuable data that can easily be integrated with glucose levels. Using CGM devices, it is possible to accurately measure important metrics, such as the outcome of a fasted MTT, the AUC of meal-induced glucose peaks, and the compliance to a fasting regimen. We explored this framework in the context of a 6-week, double-blind, placebo-controlled, 2×2 cross-over pilot study (n=6) comparing a twice daily synbiotic medical food designed to improve glucose control. We demonstrated the promise of CGM devices as a means to assess clinical outcomes of nonpharmacological interventions in rigorous studies for research purposes.

Although accurate automatic detection of glucose peaks is straightforward and can provide valuable data for the clinician and individual, how to use those peaks as clinical endpoints is challenging as the glucose peaks represent a response to real-world behaviors and not to a prequantified study-wide intervention. Commonly, diet logs are used to control for dietary variation, but they have reporting bias and increase subject burden considerably [34]. In this study, we tried to address the issues of subject burden and reporting bias by employing a smartphone app that allowed subjects to take pictures of their meals. This allows the creation of a visual diet log that is in sync with the glucose levels measured with the CGM devices, which provides a rich and valuable source of data using a solution that is simpler to implement and less burdensome to participants. However, further work is required to bridge the gap between image data and actionable carbohydrate content estimates. Variation in the regularity of eating habits or meal content could potentially lead to higher between-subject variance. Even for a within-subject contrast, the lack of regularity in eating and exercise habits increases the variance of the estimate of any treatment effect. Future studies could also leverage those types of visual logs to monitor adherence to a specific diet type (eg, high fiber diet). Complementing the photos with more precise annotations could also improve estimates. Smartphone apps that rely on drop-down menus provide fair estimates of carbohydrate consumption [35,36] and greatly outperform self-assessment [37].

On the other end of the spectrum, the MTT protocol employed in traditional clinical studies represents a very controlled setting. This test yields a much more consistent measure, but it may not reflect the real-world effectiveness of an intervention as accurately. As is expected and has been reported in various studies [38,39], compliance decreases when subjects are increasingly inconvenienced. MTTs, as required in this study, necessitated consuming a specific shake, undergoing a 6-hour fast, and monitoring for 2 to 3 hours following beverage consumption. As this disrupts an individual’s daily routine, the number of observations per subject per week needs to be limited. Focusing on breakfast-induced glucose peaks might represent a middle ground. As can be seen in Multimedia Appendix 12, all subjects, except Subject 5, had a high number of peaks at a consistent time most mornings. This denotes a regular breakfast routine at the subject level. This was confirmed in interviews, where subjects reported having either a consistent weekday breakfast or a rotation among a limited set of menus. Defining a consistent breakfast for all subjects a few days a week would provide a robust measure of the level of glucose control in a realistic setting with reduced variability.

We expect that future studies will leverage the novel capabilities of CGM devices to provide real-time compliance data. For example, a reminder to subjects whenever compliance is detected to be declining, in the form of a push notification or a phone call, could ensure that data capture remains sufficient, with the aim to improve the robustness of the inference. Subjects unanimously said in the final interviews that reasonable notifications would increase compliance, and such methods have been successfully deployed in previous studies [40]. CGM devices could also increase subjects’ engagement. In this study, subjects were presented with an interactive dashboard recapitulating their glucose and food behaviors (Figure 4). Subjects were excited about this and spent time looking at the effects of individual foods on their glucose levels. Presenting an example dashboard to the subjects before baseline, with the promise of more complete results at the end, could increase engagement in the study and therefore compliance. While such usage of CGM devices shows clear promise, care needs to be taken to ensure sound experimental design, including maintaining subject and researcher blinding to the arm assignment. In spite of these issues, proper usage of CGM devices can provide just-in-time actionable measures of compliance.

The CGM devices deployed in this study measured glucose-related metrics, but they can be complemented with other types of data collection depending on the setting. In this exploratory study, stool samples were collected. While collection of this type of data is feasible at home, the samples still need to be delivered to a clinic for analysis. However, the difficulties in collecting this type of data are limited by the fact that they are not needed for all patients or in confirmatory studies. CGM devices can also be used in coordination with other sensors (eg, smartwatches and mobility data from the smartphone) that could be complementary with little added burden on the patient [41,42]. The general principles of compliance measurements discussed above in the context of CGM devices are also applicable to any other metric relying on a connected device. Privacy concerns should also be addressed properly, as most subjects raised this issue during the interviews.

### Limitations

Previous studies [43] have linked the regularity of CGM device scanning to improvements in the “time in range” endpoint (glucose level between 70 mg/dL and 180 mg/dL). While we did not observe it in this study given the general lack of glucose intolerance in the study subjects, differences in engagement between subjects could be linked to differences in outcomes in larger studies. Controlling for adherence, directly measured through scanning and recording annotations, could limit this phenomenon and lead to less noisy estimates of the treatment effect.

Given the scale of the study, a cross-over design was employed as a means to guard against a number of potential confounders. In particular, anecdotal observations while developing the app suggested that individuals responded to the sensor in a distinct and consistent manner. Coupled with the fact that the MTTs were self-administered, a cross-over design seemed logical. Despite these factors, cross-over designs can be of limited utility in the presence of carryover or spillover effects. Indeed, strains were detected at the beginning of the placebo period in some subjects crossing over from the active product period, indicating the potential for carryover effects. No strains were detected at the end of the placebo period for those same subjects, suggesting that a longer washout period will be required in a future cross-over study.

### Conclusions

We have shown that CGM devices can provide high-quality measurements of relevant endpoints and can enable granular monitoring of subject compliance with low clinical overhead. While this was a small pilot study, we observed a meaningful effect of the study product in subjects with diminished initial glucose control. A broader follow-up study, both in size and duration, is necessary to validate our very preliminary results.

## Appendix

Multimedia Appendix 1 Supplementary methods.

Multimedia Appendix 2 Screenshot of the app.

Multimedia Appendix 3 Screenshot of the app.

Multimedia Appendix 4 Number of days spent in each period. Each subject spent at least 2 weeks in each arm of the study. The washout period between the two arms is at least 3 days.

Multimedia Appendix 5 Difference between fingerstick values and continuous glucose monitoring (CGM) estimates. Each panel shows the fingerstick value logged through the mobile app (with a red dot) and the glucose levels as measured with the CGM devices. Values are in accordance with expected values [14].

Multimedia Appendix 6 Interview guide.

Multimedia Appendix 7 According to the recommendations of Schnell et al [30], we report all hypoglycemic events, defined as excursions below 70 mg/dL for at least two consecutive values.

Multimedia Appendix 8 According to the recommendations of Schnell et al [30], we report four summary statistics at the subject level, measuring time out of range (as number of continuous glucose monitoring scans per day), and glucose variability, reported as the average within-day standard deviation of glucose levels and the standard deviation of the average daily glucose level.

Multimedia Appendix 9 Interview summary.

Multimedia Appendix 10 Area under the curve (AUC) during the self-administered meal-tolerance tests. Incremental AUCs are computed using the glucose levels provided by the continuous glucose monitoring devices. Subjects recorded the time of the ingestion of the Boost drink using the smartphone app. Incremental AUCs are rounded to the nearest unit.

Multimedia Appendix 11 Fecal quantitative polymerase chain reaction measures of formulation strain concentration. Each subject, except for Subject 4, provided stool samples. As expected, strains in the formulation were detected when the subjects were taking the formulation and not detected when the subjects were not (ie, at baseline, during placebo, and during placebo washout). Some carryover is observed. Strains are detectable during a few days following the product period.

Multimedia Appendix 12 Times of the tops of the peaks during the day. Peaks are detected using the algorithm described in Multimedia Appendix 1. For most subjects, a clear trend in the morning suggests a routine linked to breakfast.

Multimedia Appendix 13 CONSORT-EHEALTH checklist (V 1.6.1).

## Abbreviations

AUC: area under the curve
CGM: continuous glucose monitoring
MTT: meal-tolerance test
qPCR: quantitative polymerase chain reaction
RCT: randomized controlled trial
T2D: type 2 diabetes
