# Supplementary material for: Medical Food Assessment Using a Smartphone App With Continuous Glucose Monitoring Sensors: Proof-of-Concept Study
Source: JMIR Form Res. 2021 Mar 4;5(3):e20175. doi: 10.2196/20175 (PMC7974765; doi:10.2196/20175)
Supplement: Multimedia Appendix 1 [file formative_v5i3e20175_app1.pdf]

Supplementary methods for *Medical Food Assessment Using a Smartphone App With Continuous Glucose Monitoring Sensors: Proof-of-Concept Study*

1 Prepossessing and Data Normalization

The Libre sensors used in this study had a maximum lifespan of 14-days with a subset of these limited to 10-days. The sensors produce unreliable glucose concentration data for the first 6-24 hours of their use. CGM are less accurate in the first (and sometimes second) day; this inaccuracy has been attributed to the host-immune response to the application of the sensor (see below) Figure 1). Sensors are also likely to have reduced performance in the last day probably due to collagen formation around the sensor (see paragraph *Accuracy* in [1]). For these reasons, the CGM data from the first (and sometimes second) and last day of the sensors were not used in most statistical analyses.

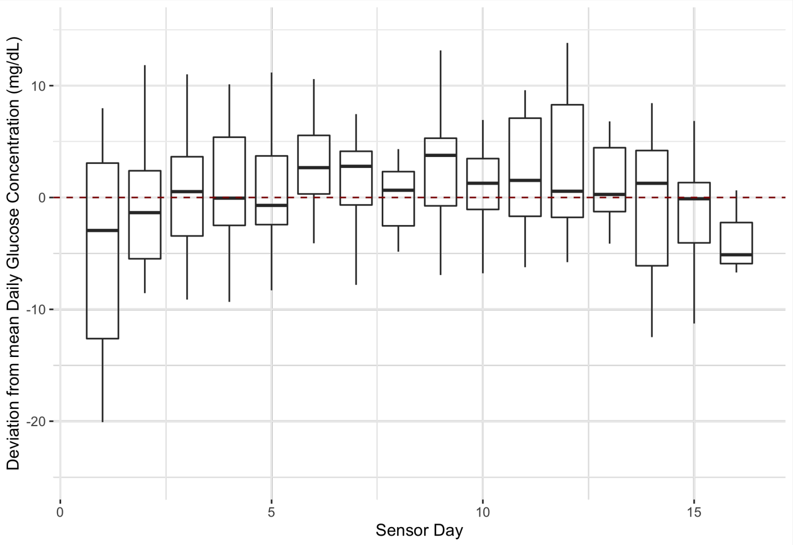

Figure 1: *Sensor age.* First and last day of a sensor were removed from the analysis because CGM underestimate glucose concentration during those days. One hypothesis is that the sensors cause local inflammation during the first (and sometimes second day), and that collagen starts forming around the sensor during the last day.

2 Product

Four out of the five synbiotic strains (*Akkermansia muciniphila*, *Eubacterium hallii*, *Clostridium beijerinckii* and *Clostridium butyricum*) were manufactured at Pendulum Therapeutics according to current Good Manufacturing Practices (cGMP) for food products in an FDA registered food manufacturing facility. Those strains were each grown in 2000-litre anaerobic batch cultures using media free of animal products and then harvested mid-log phase by centrifugation. Afterwards, individual strains were mixed to obtain the final formulation, which was then put into the capsules. The complete characterization of these strains along with data from preclinical rat toxicity and human-safety pilot studies were combined into a Generally Recognized as Safe (GRAS) dossier

which was reviewed and accepted by an independent third party (Ramboll Environ, Arlington, VA).

The four strains described above were also mixed with the commercially available strain B. infantis, purchased from Nutraceutix (Redmond, WA) as lyophilized powder. Combined study product was encapsulated in acid-resistant opaque capsules and stored at 4° C until use. The formulation contained between 9.0×10^8 and 1.6×10^10 Active Fluorescent Units (AFU), a quantitative measure of viable-cell count, of each of the specified bacteria. The placebo study product was composed of colloidal silicon dioxide (CSD). All participants were instructed to consume 3 capsules of their assigned study product twice daily prior to consuming their morning and evening meals.

3 Fecal Microbiome Analyses

All but one participant agreed to provide stool samples. Whole stool was collected by participants using a Commode Specimen Collection System (Biomedical Polymers, Sterling, MA). Upon collection, subjects were instructed to immediately freeze (home freezer, approximately -20 °C) their stool samples within a sponsor-provided thermally-insulated container. After returning to Pendulum Therapeutics, samples were kept for long-term storage at -80 °C. Stool samples were first thawed for homogenization, which consisted of: (1) suspension in a 2:1 ratio (m/m) of 50 mM Tris HCl buffer (pH 8.0) with 5 mM EDTA, (2) homogenization for five minutes in a paddle stomacher (Stomacher 3500, Seward, UK), (3) filtration through a coarse 280 μm mesh, and (4) preparation of 1 mL aliquots subsequently refrozen and maintained at -80 °C.

4 Measuring anthropometric characteristics

Weight (W) was measured with Etekcity Stainless Steel Digital Body Weight Bathroom Scale, Step-On Technology. Blood Pressure (BP) and Heart Rate (HR) were measured with iProvèn BPM-2244B. O2 levels measured with Santamedical Generation 2 SM-165 Fingertip Pulse Oximeter.

5 Power calculation

We want to estimate the sample size of a potential similar future study. In the context of using a one sided t-test, we want to test if the mean $\Delta\Delta AUC$ is negative. The Null and alternative hypotheses are

$$H0: \quad \Delta\Delta AUC < 0$$
$$H1: \quad \Delta\Delta AUC \geq 0$$

Then, formulas to compute sample size n and power $1-\beta$ are

$$n = (\sigma \frac{z_{1-\alpha} + z_{1-\beta}}{\Delta\Delta AUC})^2$$

$$1 - \beta = \phi(z - z_{1-\alpha}) + \phi(-z - z_{1-\alpha})$$

where

$$z = \frac{\Delta\Delta AUC}{\frac{\sigma}{\sqrt{n}}}.$$

With $\Delta\Delta AUC = 1550$ and $\sigma = 1568$, if we take $\alpha = 0.05$, $1 - \beta = 0.9$ (power), the estimated sample size is 37.

6 Peak detection

Consider a set X of T equally spaced points $\{x_1, x_2, ..., x_T\}$. We want to find the peaks in this time series.

6.1 Finding peak tops

The peak detection algorithm follows [2]. Two parameters are user-defined: k, which determines how wide the neighborhood around the peaks is, and h, which determines how high peaks have to get. In particular, a smaller h value lead to more peaks being selected but since those are later filtered, the algorithm is relatively insensitive to the choice of h. We pick $k = 7$ and $h = 1.5$, based on visual results.

- For each point x_i, compute $S(x_i, k) = \frac{max(x_i - x_{i-1}, x_i - x_{i-2}, \ldots, x_i - x_{i-k}) + max(x_i - x_{i+1}, x_i - x_{i+2}, \ldots, x_i - x_{i+k})}{2}$.
- Define peaks as points where $S(x_j) - \overline{S(x_i)} > h * sd_i(S(x_i))$.
- If two peaks are 30min or less apart, only keep the one with the highest S value.

6.2 Extending peaks

Start of the peak

Define the neighborhood Starting from the top of the peak i, we extend the neighborhood to the points before, stopping if the end of the previous peak is encountered, the rate is smaller than -5, the rate is negative for the second time or k points have been added. We name K the set of indexes of the left-neighbors of the peak i.

Find the start of the peak We define $j_{start} = argmin_{j \in K} x_j$. If $j_{start} \neq min(K)$, then the peak start at $x_{j_{start}}$. Else, the peak is extended on the left until $j = 1$, we encounter the end of the previous peak or the rate decreases. The last point reached is the start of the peak i.

End of the peak

Define the neighborhood Starting from the top of the peak i and we extend the neighborhood to the points after, stopping if we encounter the top of the next peak, the rate is larger than $+5$, the rate is positive for the second time or we have added k points. We name K the set of indexes of the right-neighbors of the peak i

Find the end of the peak We define $j_{end} = argmin_{j \in K} x_j$. If $j_{end} \neq max(K)$, then the peak ends at $x_{j_{start}}$. Else, we extend the peak on the right until $j = T$, we encounter the end of the previous peak or the rate increases. The point where we stop is the end of the peak i.

6.3 Merging peaks

We define a threshold *thresh*. In practice, we pick *thresh*= 20. The threshold is the minimum height of a peak, but also informs the merging criterion.

Let's consider two consecutive peaks i_1 and i_2. If $i_2 - i_1 \leq k$, that is the two peaks are less than k points apart, and $min(x_{i_1}, x_{i_2}) - min_{i_1 < j < i_2}(x_j) < \frac{thresh}{2}$, that is the glucose drops by less than $\frac{thresh}{2}$ between the two peak tops, then the peaks were merged. The new peak starts at the start of peak i_1, ends at the end of peak i_2, and peaks at $i_{top} = argmax_{i \in \{i_1, i_2\}} x_i$.

6.4 Filter peaks

A peak i is filtered out if $x^{(i)}_{top} - min(x^{(i)}_{start}, x^{(i)}_{end}) < thresh$

References

[1] Daniel DeSalvo and Bruce Buckingham. Continuous glucose monitoring: current use and future directions. *Current diabetes reports*, 13(5):657–62, oct 2013. ISSN 1539-0829. doi: 10.1007/s11892-013-0398-4. URL http://www.ncbi.nlm.nih.gov/pubmed/23943230http://www.pubmedcentral.nih.gov/articlerender.fcgi?artid=PMC5164922.

[2] Girish Palshikar. Simple Algorithms for Peak Detection in Time-Series Social Media Analysis View project Human Safety View project Simple Algorithms for Peak Detection in Time-Series Simple Algorithms for Peak Detection in Time-Series, 2009. URL https://www.researchgate.net/publication/228853276.
