# Supplementary material for: Medical Food Assessment Using a Smartphone App With Continuous Glucose Monitoring Sensors: Proof-of-Concept Study
Source: JMIR Form Res. 2021 Mar 4;5(3):e20175. doi: 10.2196/20175 (PMC7974765; doi:10.2196/20175)

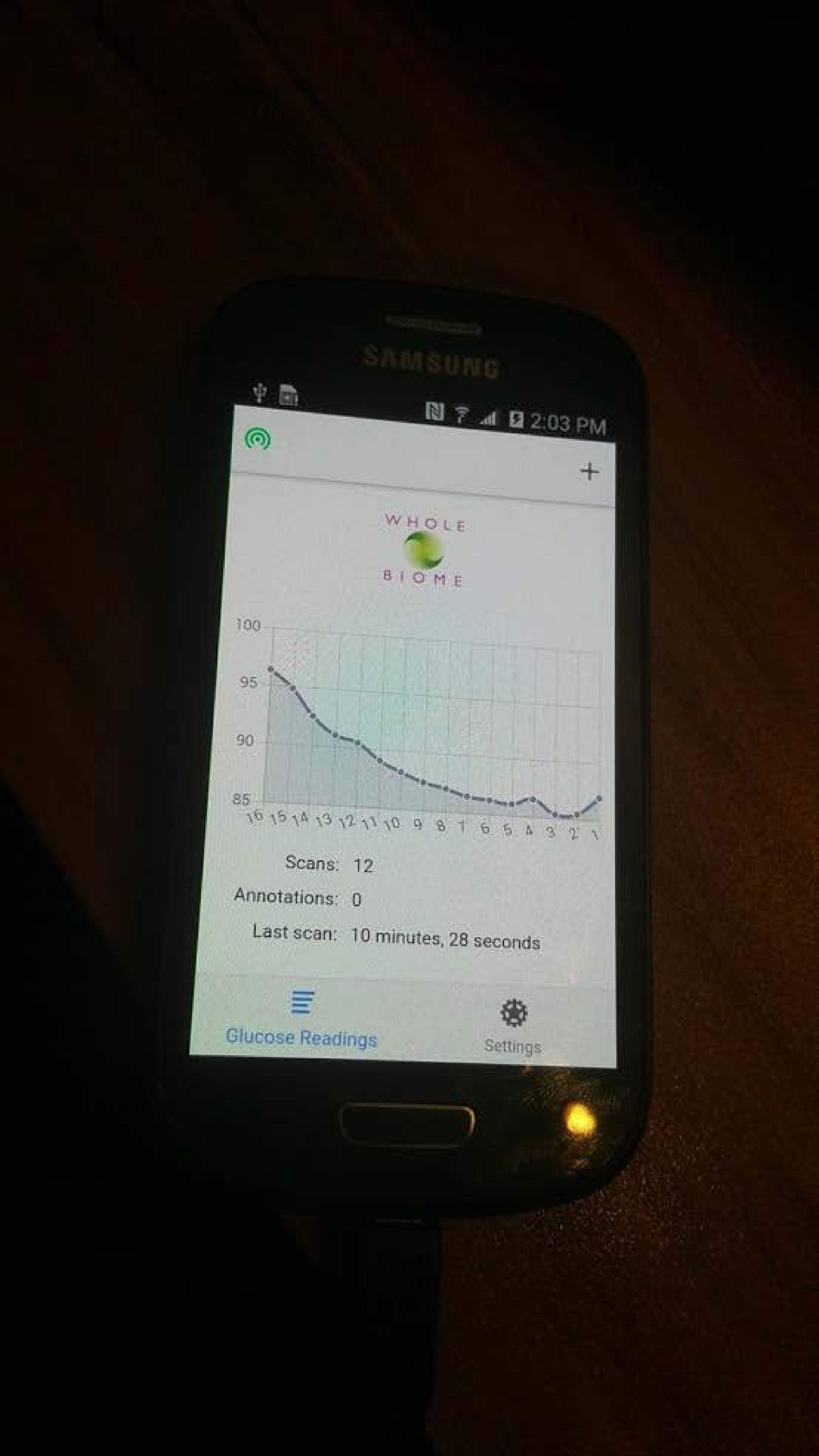
SAMSUNG
2:03 PM
WHOLE
BIOME
100
95
90
85
16 15 14 13 12 11 10 9 8 7 6 5 4 3 2 1
Scans: 12
Annotations: 0
Last scan: 10 minutes, 28 seconds
Glucose Readings
Settings

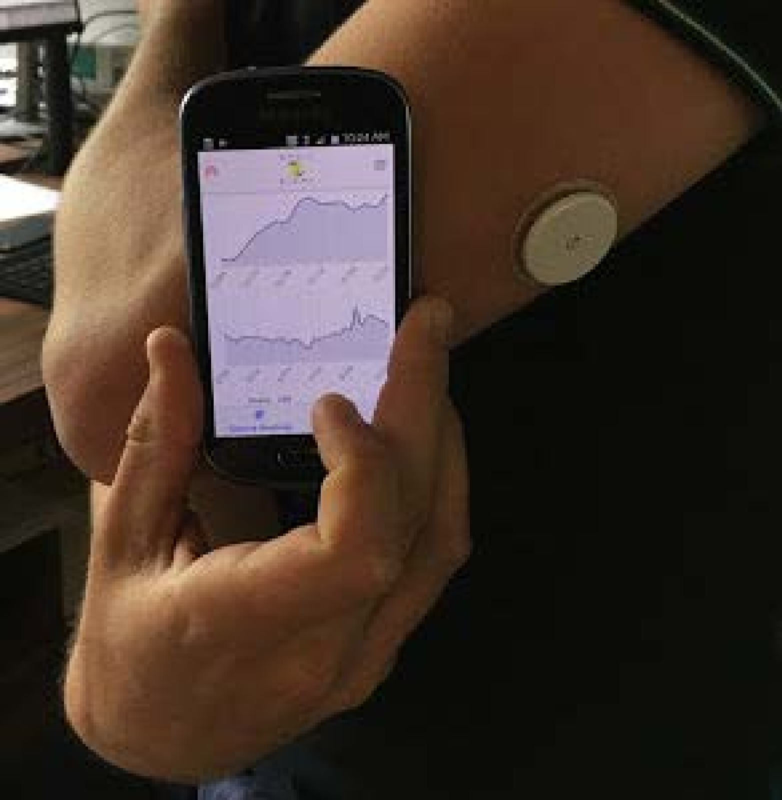

Supplement: Multimedia Appendix 2 [file formative_v5i3e20175_app2.pdf]
