# Supplementary material for: Medical Food Assessment Using a Smartphone App With Continuous Glucose Monitoring Sensors: Proof-of-Concept Study
Source: JMIR Form Res. 2021 Mar 4;5(3):e20175. doi: 10.2196/20175 (PMC7974765; doi:10.2196/20175)
Supplement: Multimedia Appendix 3 [file formative_v5i3e20175_app3.pdf]

W H O L E
B I O M E

Scans: 143

Last scan: 1 hour, 2 minutes, 56 seconds

Glucose Readings

Food Log

New Annotation

×

Description

ADD PHOTO

CREATE ANNOTATION
