# Supplementary figures and images for: Medical Food Assessment Using a Smartphone App With Continuous Glucose Monitoring Sensors: Proof-of-Concept Study
Source: JMIR Form Res. 2021 Mar 4;5(3):e20175. doi: 10.2196/20175 (PMC7974765; doi:10.2196/20175)

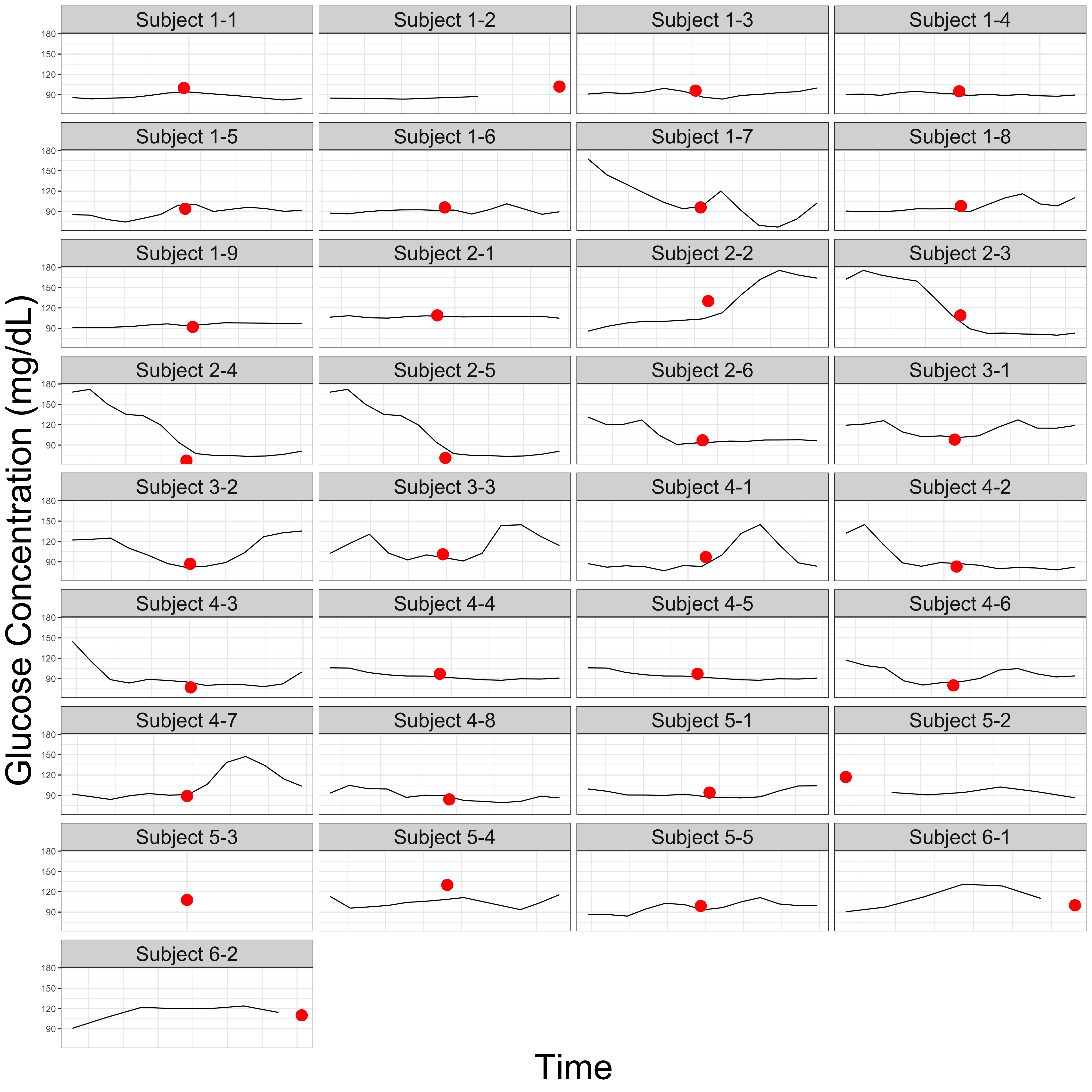

Supplement: Multimedia Appendix 5 [file formative_v5i3e20175_app5.png]

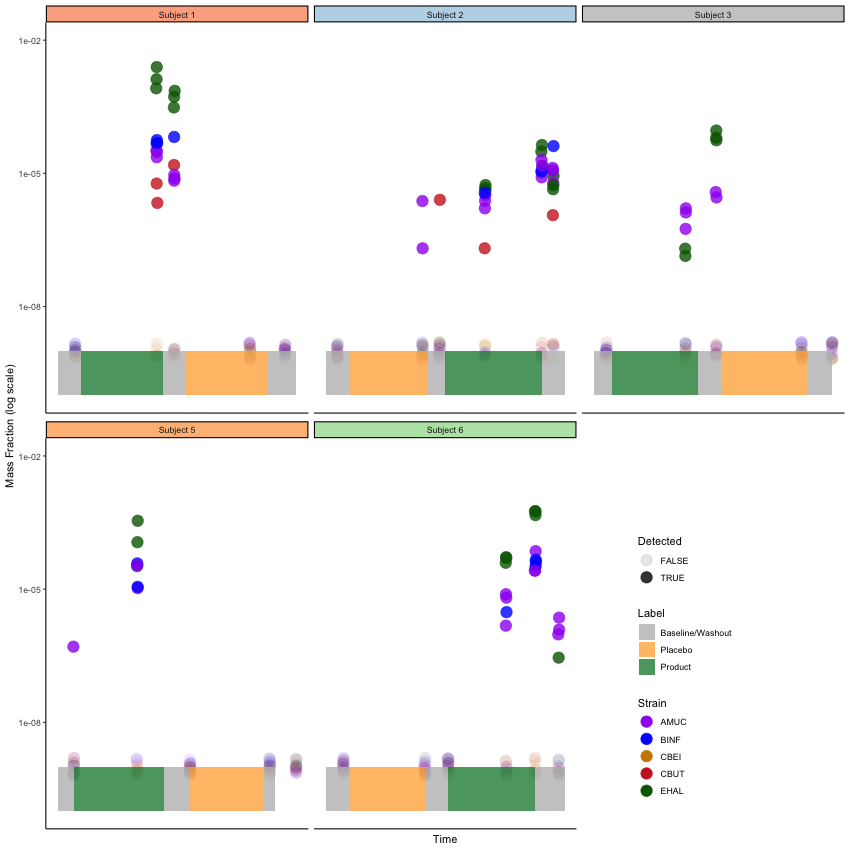

Supplement: Multimedia Appendix 11 [file formative_v5i3e20175_app11.png]

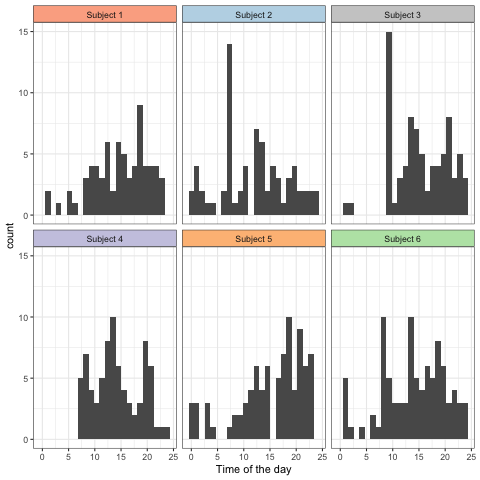

Supplement: Multimedia Appendix 12 [file formative_v5i3e20175_app12.png]
