# Supplementary material for: Medical Food Assessment Using a Smartphone App With Continuous Glucose Monitoring Sensors: Proof-of-Concept Study
Source: JMIR Form Res. 2021 Mar 4;5(3):e20175. doi: 10.2196/20175 (PMC7974765; doi:10.2196/20175)
Supplement: Multimedia Appendix 6 [file formative_v5i3e20175_app6.pdf]

Interview Guide

Design

- What did you like / notice in the overall study?
- What did you NOT like / notice in the overall study?
- How did you feel in leg 1?
    - Any major changes?
    - What did you think you were on? Placebo or product? Why
- How did you feel in leg 2?
    - Any major changes?
    - What did you think you were on? Placebo or product? Why

Product

- Issues or comments on
    - Storing product
    - Taking product
    - Morning vs Night consumption
    - Need to take product twice a day
    - Number of capsules required
- Anything objectionable about the product?
- Based on your experience, would you be willing to continue using the product on a constant basis?
- Have you noticed change in mass/volume or characteristics of your stool?
    - During or after the study periods?
    - During the study?
    - During a specific period of the study?
    - During or after the second wash-out period?
    - If you did notice changes, were they reflected in the previous stool samples collected?

CGM

- What worked / didn't work with putting it on?
- Did it hurt?
    - When you put it on?
    - Wear it?
- Any issues?
    - When putting / taking off a shirt?
- What did you learn from the data?
- Other learning?
- Any Surprises?

App

- Taking readings:
  - observations?
  - What worked?
  - What didn't?
- Food diary:
  - observations?
  - What worked?
  - What didn't?

Boost

- Does the date and time of the boost test coincide with the date and time listed above?
  - If the listed information is not correct, what changes should be made?
- Preceding each BTT, how long did you fast?
  - For at least 5 hours?
  - Coffee? Sugar? With milk/sugar?
- Preceding or during each MTT
- Did you physically exert yourself, e.g., jogging, walking to work, etc? If yes, what was the exercise?
- Did you take a picture of the boost immediately before consuming the boost, if not, can you remember approximately when in relation to the picture you took the boost? Did you consume the boost in 1 minute, 10 minutes, 1 hour?

Behavior changes

- What behaviors changed for you?
  - Eating changes?
  - Mood changes?
  - Behavioural changes?
  - Social changes?
- How did this work
  - with others around?
  - In social settings?
  - when going out to eat?
- Sleep
  - Is your sleep schedule regular?
  - Did the character of your sleep change at any point is the study?
  - Do you remember any instances during the study that you slept poorly, awoke during the night, etc.?
  - Is there a child in the house that necessitates you being up during the night?
  - Do you remember any instances during the study that you ate during the night?

- How frequently do you eat the same meal for breakfast?
    - Were there any study days where you had a breakfast that differed significantly from your routine?
    - During the study were there any occasions where you had a meal that was much larger than usual? If so, when did it occur.
    - Are there any evenings during the study where you had more alcohol that usual?
- Sport: Do you remember any instances during the study that you physically exert yourself more than usual, e.g., long jogging, biking to work when late for a meeting, etc.
- Did you feel less excitement by the end of the study?
- Did it affect the frequency of your scans, missed dose?
- Ideas to keep excitement along the study?

Future Studies

- Would you mind tracking activity data
- Would you have been more compliant if compensation was increased according to compliance
- Would notifications/alerts during the study to remind you to scan, fast, etc. be helpful, annoying, ...?
- How much would your compliance have been affected by having the application on your personal cell phone?
- What information could be provided via the app to encourage usage, e.g., daily/hourly/weekly summaries? Recent meals?

Closing

- Would you do another study similar to this one?
- Would you recommend others to a study like this? On a scale of 1-10 with 1 being no and 10 being absolutely
