# Supplementary material for: Medical Food Assessment Using a Smartphone App With Continuous Glucose Monitoring Sensors: Proof-of-Concept Study
Source: JMIR Form Res. 2021 Mar 4;5(3):e20175. doi: 10.2196/20175 (PMC7974765; doi:10.2196/20175)
Supplement: Multimedia Appendix 9 [file formative_v5i3e20175_app9.pdf]

Feedbacks on Product and Application

Overall

- Everyone would participate in the study again and all would highly recommend to other to be a part of a study like this in the future (scores ranging from 7/10 to 10/10 with mean at 8.25).
- The most consistent difference in the product vs. placebo relates to stool quality. It is not clear whether the product provides “better” stool or the placebo causes constipation
- CGM was seen as a positive with few minor issues (e.g. ripping off due to taking off a shirt)

Product and packaging

- Current packaging (snap top) was neutral to positive. Product smell was a plus. However, one person complained that the sachets “got in the way” when getting the product
- Taking 3 pills at each use was seen as neutral, however, one person did have some issues swallowing
- Pill size was seen as neutral to negative (i.e. too big) and is likely linked to 3 pill requirement since most people took 3 at a time (see habits with product)
- Storing the product in the refrigerator was not an issue (but see issue with evening compliance)
- There was mixed feedback on whether people thought they had more energy (e.g. “sprint in my step") when on product

Habits with product

- Most people take all three pills in one swallow (vs. taking one at a time)
- Most people take the pills with water
- Most people found it easier to remember to take pills in the morning easier to integrate into routine and less likely to forget to take the product (vs. evening)
- More likely to take while prepping meal or while eating the meal
- The variability of dinner routines, timing and more social settings made the evening habit more difficult with compliance
- Because of refrigeration, eating out (dinner) made it harder to take at or within 30 minutes of the meal as directed
- Numerous situations (linked to eating out) caused people to take the product before going to sleep. This may be related to poor sleeper quality for one participant.

CGM

- Overall, everyone enjoyed seeing the real time feedback from the CGM and thought the CGM was a positive experience
- The vibration confirmation on the scan was very positive
- However, the app did crash a lot and require multiple tries for a scan upload
- The vast majority of the CGMs were applied by someone else in the clinic.
- It is not clear what issues will arise with personal application with no one else present (e.g. proper application, proper location, etc.).This is a specific area that should be studied before a future study

- Only one person required additional adhesive (black tape) due to the person's workout type and intensity. Two people had the CGM rip out because of clothing snags: this is a clear watchout for future studies. Two people had minor soreness with the CGM, one surmised that they hit a muscle, one likely correlated with inflammation on day one of a new sensor

The app

- People did not like having two phones but for a study it was manageable
- The majority of the participants were iPhone users, but the android phone was more than manageable
- The sequence of operations for annotating + picture taking + submitting was not clear and caused confusion
- Most participants were less likely to take pics in social setting and thus annotated after the fact (e.g. “I ate xxx 90 minutes ago”). However, instagram has lowered this barrier
- All participants would have liked to see previous images and annotations.
- Uploading and being on wifi for the app was not clearly understood and required multiple follow ups from study administrators
- All people agreed that reminders / alerts would help (within reason)

Personal learning

- All participants learned from the real time feedback of eating something and seeing what happened with their curves
- Documenting / tracking food definitely increases awareness and likely causes positive choices over time
- Most wanted to see the data on an ongoing and historical basis. “Once you took pic and submitted you couldn’t access it”
- Most people focused on the size of the spike vs. on the time it takes to get back to normal levels
- All people wanted to see the actual numbers; some learned of the hack (holding down on the graph); and some used finger pricks to self calibrate
- All participants shared feedback on wanting “more data, more insights”

Study design

- All participants experienced fatigue / novelty wearing off as the study proceeded
- Those who thought they were on product on the 2nd leg may have been more motivated
- Gamification and competition (e.g. points, leaderboards, etc.) were the overwhelming recommendations on ways to improve future study designs
- Compensation was positive and most were not expecting it; this population was not motivated by the compensation
- Future studies that add Fitbit type data was seen as positive and manageable
- Some privacy concerns were brought up with anything associated with GPS (even though the phone itself likely tracked this) and anything beyond fitbit type data
- We did not probe on stool sampling and the boost test but it is likely known that both can be improved.
